# PIMT Prevents the Apoptosis of Endothelial Cells in Response to Glycated Low Density Lipoproteins and Protective Effects of Grape Seed Procyanidin B2

**DOI:** 10.1371/journal.pone.0069979

**Published:** 2013-07-26

**Authors:** Xiao-li Li, Bao-ying Li, Mei Cheng, Fei Yu, Wen-bin Yin, Qian Cai, Zhen Zhang, Jian-hua Zhang, Jun-fu Wang, Rui-hai Zhou, Hai-qing Gao

**Affiliations:** 1 Key Laboratory of Cardiovascular Proteomics of Shandong Province, Department of Geriatric Endocrinology, Qi-Lu Hospital of Shandong University, Jinan, China; 2 Department of Drug Purchase and Supply, Qi-Lu Hospital of Shandong University, Jinan, China; 3 Institute of Basic Science, Medical Science Academy of Shandong, Jinan, China; 4 Division of Cardiology, University of North Carolina at Chapel Hill, Chapel Hill, North Carolina, United States of America; Bambino Gesu' Children Hospital, Italy

## Abstract

**Background:**

The development of diabetic angiopathy is associated with profound vascular endothelial cells (VEC) dysfunction and apoptosis. Glycated low density lipoproteins (gly-LDL) continuously produced in the setting of diabetic patients play an important role in causing VEC dysfunction and apoptosis. However, the underlying molecular mechanism remains largely elusive. Protein L-isoaspartyl methyltransferase (PIMT) is a widely expressed protein repair enzyme by multiple cell types of arterial wall including VEC. Our previous proteomic studies showed that the expression of PIMT was significantly decreased in the aorta of diabetic rats as compared with control rats and treatment with grape seed procyanidin extracts significantly increased the PIMT expression in diabetic rats. We hypothesized that PIMT plays a critical role in gly-LDL induced VEC apoptosis; grape seed procyanidin B2 (GSPB2) protect against gly-LDL induced VEC apoptosis through PIMT regulation.

**Methods and Results:**

HUVEC transfected negative control and PIMT siRNA were treated with or without GSPB2 (10 µmol/L) for 48 h. Moreover, HUVEC of PIMT overexpression were stimulated by gly-LDL (50 µg/ml) in the presence or absence of GSPB2 (10 µmol/L) for 48 h. Our results showed that gly-LDL downregulated PIMT expression and PIMT overexpression or GSPB2 significantly attenuated gly-LDL induced VEC apoptosis. PIMT siRNA increased VEC apoptosis with up-regulation of p53, cytochrome c release, caspase-9 and caspase-3 activation. Mechanistically, overexpression of PIMT or GSPB2 increased the phosphorylation of ERK1/2 and GSK3β in the gly-LDL induced VEC.

**Conclusion:**

In summary, our study identified PIMT as a key player responsible for gly-LDL induced VEC apoptosis and GSPB2 protect against gly-LDL induced VEC apoptosis by PIMT up-regulation. Targeting PIMT including use of GSPB2 could be turned into clinical application in the fighting against diabetic vascular complications.

## Introduction

Vascular complications remain a leading cause of morbidity and mortality in subjects with diabetes, and experimental evidence suggests that progression of diabetes is associated with profound endothelial dysfunction [1]. The endothelium is the active inner monolayer of the blood vessels, forming an interface or barrier between circulating blood in the lumen and the rest of the vessel wall. It plays a very important role in maintenance of vascular integrity by protecting the vessels from activation of clotting and proinflammatory factors. An imbalance in repair and injury resulting in early vascular changes, including endothelial dysfunction and apoptosis, can be seen in both experimental diabetic animal models and patients [2], [3]. Endothelial dysfunction and apoptosis have thus emerged as an important early target for preventing atherosclerosis and cardiovascular disease in the diabetes mellitus (DM).

Hyperglycemia and hyperlipidemia are important factors in the development of endothelial dysfunction and apoptosis in the DM. Low density lipoproteins (LDL) glycation is increased in diabetic patients because of their elevated plasma glucose concentrations [4]. Glycated LDL (gly-LDL) has been implicated in diverse processes: triggering apoptosis, reducing nitric oxide synthesis, and impairing vascular reactivity [5]. In vascular endothelial cells (VEC), gly-LDL increased oxidative stress leading to cellular dysfunction and apoptosis. However, the molecular mechanism underlying gly-LDL induced endothelial dysfunction and apoptosis remains elusive, which has been the bottle neck in the development of effective therapeutic approaches to the prevention and treatment of diabetic vascular complications [6], [7].

Protein L-isoaspartyl methyltransferase (PIMT) is a widely expressed protein repair enzyme by limiting the accumulation of altered aspartyl (L-isoaspartyl) residues in proteins. The formation of L-isoaspartyl residues is a spontaneous process that is related to aging and may lead to loss of protein function [8]. The expression of PIMT has been found in all tissues examined in rat. It is also highly expressed in human beta-cells, endothelial cells and smooth muscle cells [9], [10]. Our previous proteomic studies showed that the expression of PIMT in the aorta of diabetic rats was significantly lower than those of control rats. We also found that treatment with grape seed procyanidin extracts (GSPE) significantly increased the expression of PIMT in diabetic rats [11]. Research to date has suggested that PIMT is linked to apoptosis, which is an essential cellular process for cell death [12]. Recent studies have shown that the induction of PIMT protects cells from apoptosis caused by H_2_O_2_ stress, and blocks the formation of reactive oxygen species, suggesting an antioxidant function of this enzyme [13].

GSPE derived from grape seeds have been reported to possess anti-oxidant, anti- nonenzymatic glycosylation, anti-inflammation, and anti-tumor effects [14]–[16]. Dimeric procyanidin B2 is one of the main components of GSPE, composed of two molecules of the flavan-3-ol (-)-epicatechin linked by a 4b→8 bonds. Several studies have shown that procyanidin B2 exerted a variety of anti-inflammatory, anti-tumor effects greater than other dimers, such as procyanidins B1, B4, and B5, at the same concentrations [17]. Our previous data showed that GSPB2 could inhibit human umbilical vein endothelial cell (HUVEC) apoptosis and exert protective effect on the development of atherosclerosis in DM [18].

The aim of the present study was to clarify the molecular mechanism underlying gly-LDL induced endothelial cell apoptosis and protective effects of GSPB2. Knockdown or overexpression of PIMT in HUVEC was used to investigate the role of PIMT in apoptosis. We also further investigated the signaling cascade involved PIMT in apoptotic process.

## Materials and Methods

### Materials

GSPB2 (>95% purity, Lot No: 20110120) was purchased from Jianfeng Inc (Tianjin, China). Bovine serum albumin (BSA), D-glucose, collagenase, trypsin/EDTA solution, dimethyl sulfoxide (DMSO), and 3-(4,5-dimethylthiazol)-2, 5-diphenyl tetrazolium bromide (MTT) were purchased from Sigma (St. Louis, USA). Fetal bovine serum (FBS) and RPMI 1640 were obtained from GIBCO (Grand Island, USA). HUVEC were obtained from American Type Culture Collection (Rockville, USA). LDL was purchased from Yiyuan biotechnology (Guangzhou, China). Terminal deoxynucleotidyl transferase biotin-dUTP nick end labeling (TUNEL) in situ apoptosis detection kit (Roche Diagnostic, Indianapolis, IN). Cytochrome c enzyme-linked immunosorbent assay kit was purchased from eBioscience (San Diego, USA). The activity kit of caspase-9 and caspase-3 were purchased from R&D Systems (Minneapolis, USA). The antibody of PIMT and P53 were purchased from Abcam (Cambridge, USA). The antibodies of glycogen synthase kinase 3β (GSK3β), phospho-GSK3β, extracellular regulated protein kinases 1/2 (ERK1/2) and phospho-ERK1/2 were purchased from Cell Signaling Technology (Beverly, MA). All other reagents were standard commercial high-purity materials.

### Gly-LDL Preparation

LDL preparations were diluted to 2 mg of protein per milliliter with 0.1 mol/L phosphate buffer (pH 7.4) containing 0.01% EDTA and 0.01% NaN3 and then incubated with 50 mmol/L glucose and equimolar amounts of NaBH3CN for 3 weeks at 37°C in the dark under N2. Native LDLs were processed identically except without the addition of glucose. At the end of glycation, lipoproteins were dialyzed to remove free glucose [19]. The extent of glycation in glycated LDL was estimated using trinitrobenzenesulfonic acid assay. Endotoxin concentrations were measured by the limulus amebocyte lysate assay (Endos), which revealed negligible values (<0.2 µg/L). LDL and its modified forms were stored in sealed tubes under a layer of nitrogen at 4°C in the dark to prevent auto-oxidation.

### Cell Cultures

HUVEC were cultured in complete medium RPMI 1640 containing 10% FBS at 37°C in a humidified atmosphere containing 50 ml/L CO2. GSPB2 was dissolved in DMSO and diluted so that the final concentration of DMSO was <0.1%.

### Knockdown of PIMT by siRNA, Construction of PIMT Overexpression Plasmids and Transfection

Short interfering RNAs (siRNAs) for PIMT and negative control siRNAs were designed and chemically synthesized from Shanghai GenePharma Co., Ltd (Shanghai, China). The siRNA sequence targeting PIMT including: sense 5′- CUCGGAGCUAAUCCACAAUTT -3′, antisense 5′- AUUGUGGAUUAGCUCCGAGTT -3′. The sequence of negative control siRNA is: sense 5′-UUCUCCGAACGUGUCACGUTT-3′, antisense 5′-ACGUGACACGUUCGGAGAATT-3′. For transient transfection, HUVEC were cultured in six-well plates overnight and transfected with siRNA against PIMT using Lipofectamine 2000 (Invitrogen Life Technologies, Carlsbad, CA) according to the instructions of the manufacturer. The expression of PIMT was assayed 48 h after transfection by real-time PCR and western blotting.

Overexpression plasmids PIMT were constructed as follows from Shanghai GenePharma Co., Ltd (Shanghai, China). Briefly, full-length cDNA of PIMT (GenBank accesion no: NM_005389.2) was amplified from a human cDNA library (open biosystem, USA) by PCR using forward primer (5′ AGATCTATGCCGGGAGCGCGCAGTG3′) and reverse primer (5′GAATCCTCACTTCCACCTGGACCACTGC). The sequences of PIMT were confirmed by sequencing. The PCR products were then subcloned into pEGFP-C vector. The overexpression plasmids with PIMT or enhanced green fluorescent portein (EGFP) were made. HUVEC (1×106) were transduced with using Lipofectamine 2000 according to the manufacturer’s instructions. Transfectant clones were characterized using real-time PCR and western blotting for the expression levels of PIMT at 48 h.

### Cell Viability Analysis

Cell viability was measured by MTT colorimetric assay. HUVEC with overexpression or siRNA knockdown of PIMT (1×106 cells/ml) were incubated for 48 h at 37°C in the presence or absence of GSPB2 (10 µmol/L). Moreover, HUVEC transfected with PIMT overexpression plasmids were treated with 50 µg/mL of gly-LDL. MTT (0.5 mg/ml) was added at 37°C for 4 h and cell viability was measured per instruction.

### Detection of Apoptosis by TUNEL Assay

HUVEC with overexpression or siRNA knockdown of PIMT were treated with or without GSPB2 (10 µmol/L) for 48 h. The apoptotic cells were determined by TUNEL assay using an in situ apoptosis detection kit according to the manufacturer’s instructions. The number of apoptotic cells was counted in ten randomly selected fields.

### Cytochrome c Release, Caspase-9 and Caspase-3 Activity Assay

For analysis of cytochrome c release into the cytosol, subcellular fractionation was performed to separate the mitochondria from the cytosol, and only the cytosolic fraction was used in the assay. After subcellular fractionation, the cytochrome c concentration in the cytosol was measured using the ELISA kit following the manufacturer’s instructions. Absorbance was determined using a microplate reader set to 450 nm with a wavelength correction set to 540 nm. The caspase-9 and caspase-3 activity in cell lysates was measured using colorimetric assay kit, per the manufacturer’s instructions. Briefly, HUVEC were removed from culture dishes and pelleted by centrifugation. Cell pellets were then treated for 10 min with iced lysis buffer supplied with the caspase-9 and -3 assay kits. Then the suspensions were centrifuged at 10,000 g for 10 min, and the supernatants were transferred to a clear tube. The specific substrate conjugate [acetyl-Leu-Glu-His-Asp- p-nitroaniline (Ac-LEHD-p-NA) for caspase-9 and acetyl-Asp-Glu- Val-Asp-p-nitroaniline (Ac-DEVD-p-NA) for caspase-3] was added, and tubes were incubated at 37°C for 2 h. During incubation, the caspases cleaved the substrates to form p-NA. Caspase-9 and -3 activities were read in a microtiter plate reader at 405 nm. The data are expressed as the mean optical density of the samples normalized to a percentage of the control value.

### Quantitative Real-time PCR

Total RNA was extracted from cells and underwent quantitative analysis of mRNA expression of PIMT by real-time PCR with an ABI Prism 7500 sequence detection system (Applied Biosystems, UK). The reaction reagent SYBR Green I Master Mix Kit (Stratagene) was used according to the manufacturer’s instructions. First strand cDNA was synthesized from total RNA per instructino (RT kit from MBI, Canada). The real-time PCR reaction components consisted of 10 µl of 2×SYBR Green I Master Mix, 0.5 µl up-stream primers (10 µmol/L), 0.5 µl down- stream primers (10 µmol/L), 1 µl cDNA and 8 µl double distilled water. Sequences of the primer sets used were as follows: PIMT (forward) 5′- CTGAAGGTGGTTCTGTACCTGC -3′, (reverse) 5′- GGAGATTGTGGATTAGCTCCGA-3′; β-actin (forward) 5′-TGGCACCCAGCACAATGA A-3′, (reverse) 5′-CTAAGTCATAGTCCGCCTAGAAGCA- 3′. The PCR conditions for all genes were as follows: 50°C for 2 min; preheating, 95°C for 10 min; followed by 40 cycles of denaturation (95°C for 15s) and annealing/elongation (62°C for 50s). Each sample was run in triplicate. Gene-specific mRNA was normalized to β-actin mRNA as an internal control. The amounts of PIMT mRNA were expressed as fold change relative to those of untreated cells set as 1.

### Western Blotting

Equal amount of proteins were separated by SDS- PAGE (12%) and transferred onto a polyvinylidene difluoride membranes (Millipore, Bedford, MA, USA).Membranes were blocked with 5% (w/v) non-fat milk dissolved in TBST (Tris-buffered saline and 0.05% Tween-20) for 1 h at room temperature and then incubated with the blocking solution containing first antibody overnight at 4°C. After washing three times, the blot was incubated with a second antibody. Bands of identity were visualized with enhanced DAB color reagent following the manufacturer’s recommendations. Quantification of the luminosity of each identified protein band was performed using a densitometric analysis (Digital Protein DNA Imagineware, Huntington Station, NY, USA).

### Statistical Analysis

Data are expressed as mean ± standard deviation. Statistical analysis between groups was made using one-way analysis of variance (ANOVA) followed by Tukey's HSD test for multiple comparisons. *P* value <0.05 was considered statistically significant. All analyses were performed with SPSS for Windows software version 10.0 (SPSS, Chicago, USA).

## Results

### Transduction Efficiency with PIMT siRNA and Overexpression Plasmids

**Figure 1:**
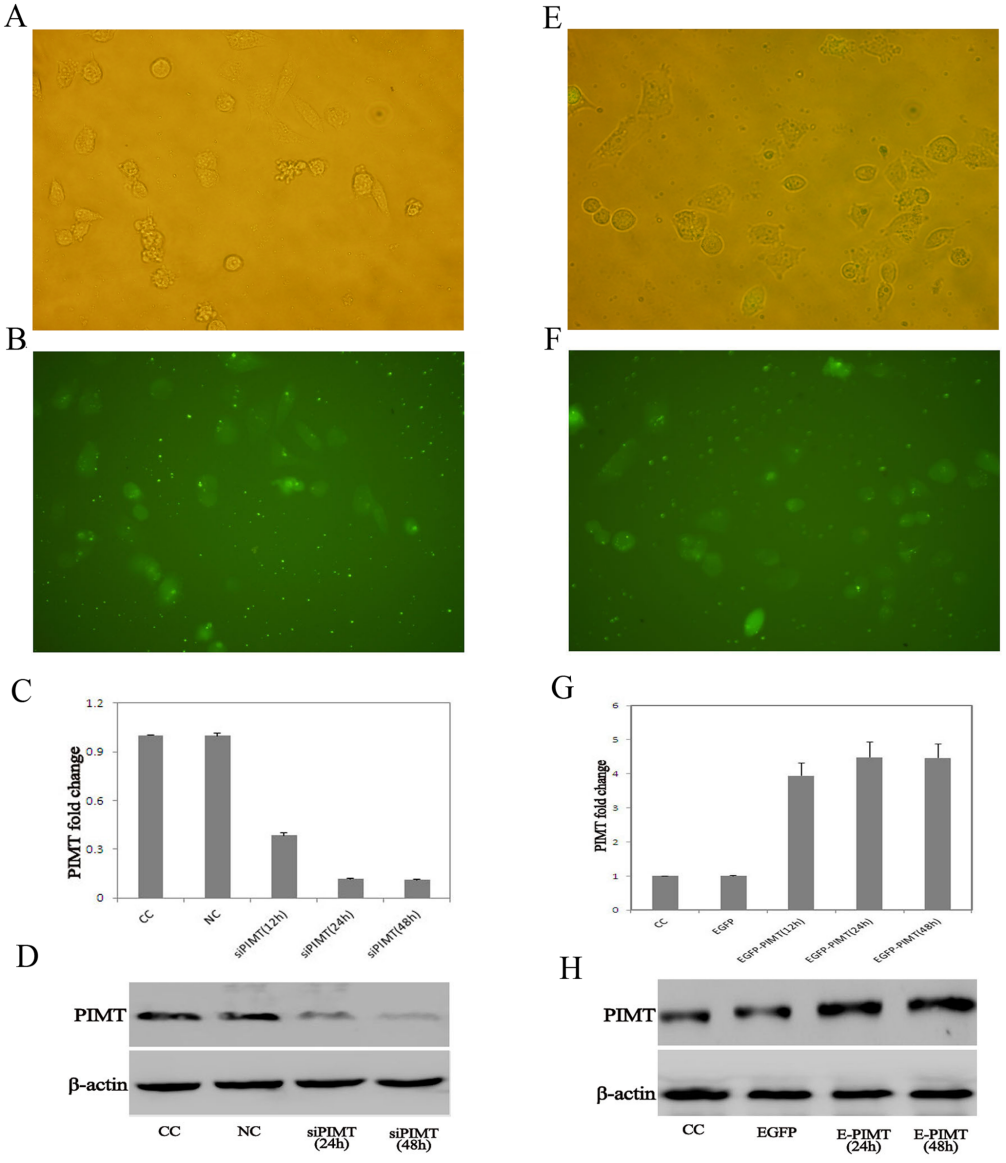
PIMT siRNA and overexpression plasmids transfection HUVEC. A, B: Bright field and fluorescence micrograph displays PIMT siRNA in HUVEC (× 200). C: RT-PCR analysis demonstrates PIMT mRNA expression in HUVEC at 12 h, 24 h, and 48 h after transfection. D: Western blot analysis demonstrates PIMT protein expression in HUVEC at 24 h and 48 h after transfection. E, F: Bright field and fluorescence micrograph displays EGFP-PIMT overexpression in HUVEC (× 200). G: RT-PCR analysis demonstrates PIMT mRNA expression in HUVEC at 12 h, 24 h, and 48 h after transfection. H: Western blot analysis demonstrates PIMT protein expression in HUVEC at 24 h and 48 h after transfection. CC group: normal control cells; NC group: negative control siRNA cells; siPIMT group: siRNA against PIMT cells; EGFP group: HUVEC carrying EGFP; E-PIMT group: HUVEC carrying both EGFP and PIMT. PIMT: protein L-isoaspartyl methyltransferase; HUVEC: human umbilical vein endothelial cells.

We transduced the HUVEC with siRNA or overexpression plasmids. Transduction conditions were optimized by using different MOI and the transduction efficiency was assessed by fluorescence microscopy, real-time PCR and western blotting. HUVEC carrying negative control siRNA (NC), HUVEC carrying siRNA against PIMT (siPIMT) and HUVEC carrying EGFP (EGFP), HUVEC carrying both EGFP and PIMT genes (E-PIMT) were harvested. The transduction efficiency was about 90% or higher at 48 h. Transgene expression was confirmed by fluorescent microscopic imaging for PIMT protein expression (Figure 1A, 1D). The protein expression of PIMT in siPIMT group decreased to more than 60% the level of the NC group at 48 h after transfection (Figure 1B, 1C). PIMT mRNA and protein expression reached its highest level at 48 h after transfection with overexpression plasmids (Figure 1E, 1F).

### Effects of gly-LDL or High Glucose on Viability in HUVEC and Treated by GSPB2

**Figure 2:**
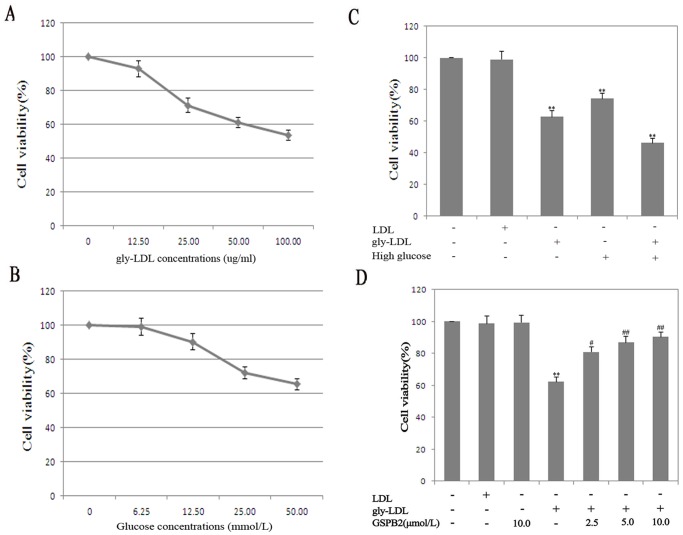
Effects of gly-LDL or high glucose on viability in HUVEC with MTT. A: Effects of different concentrations gly-LDL on viability in HUVEC at 48 h. B: Effects of different concentrations D-glucose on viability in HUVEC at 48 h. C: Effects of gly-LDL (50.00 µg/ml) or/and high glucose (25.00 mmol/L) on viability in HUVEC at 48 h. D: Effects of GSPB2 on viability in HUVEC treated by gly-LDL at 48 h. Results are expressed as percent of untreated cells (100%) and are given as mean ± SD from five independent experiments. *P<0.05, **P<0.01 compared with CC group; ^#^ *P*<0.05, ^##^ *P*<0.01 compared with gly-LDL group.

In order to assess the effects of gly-LDL or high glucose on cell growth in HUVEC, the viable cell number was measured by microscopic examination of trypan blue dye exclusion and cell survival was estimated with MTT assay. The cell viability of different concentrations gly-LDL (0, 12.50, 25.00, 50.00, 100.00 µg/mL) or D-glucose (0, 6.25, 12.50, 25.00, 50.00 mmol/L) was significantly decrease at 48 h (Figure 2A, 2B). Moreover, the cell viability was significantly lower by stimulated of HUVEC with gly-LDL (50.00 µg/mL) or/and high glucose (25.00 mmol/L) (Figure 2C). The pretreatment of HUVEC with different concentrations of GSPB2 (2.5, 5.0, 10.0 µmol/L) significantly improved the gly-LDL stimulated cell viability in a dose dependent manner for 48 h (*P*<0.05) (Figure 2D).

### Effects of gly-LDL or High Glucose on the Expression of PIMT in HUVEC and Treated by GSPB2

**Figure 3:**
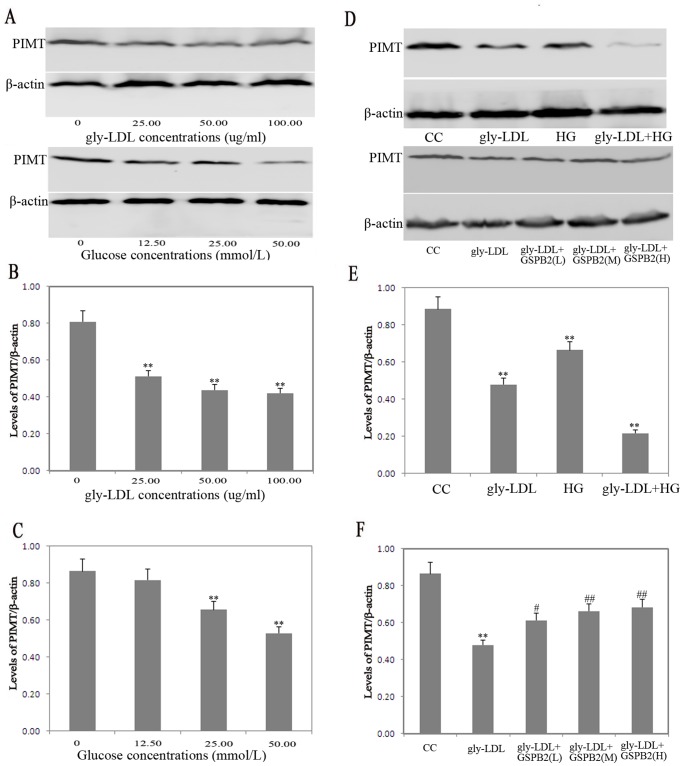
Effects of gly-LDL or high glucose on the expression of PIMT in HUVEC. A, B, C: Effects of different concentrations gly-LDL or D-glucose on the expression of PIMT in HUVEC at 48 h. D, E: Effects of gly-LDL (50.00 µg/ml) or/and high glucose (25.00 mmol/L, HG) on the expression of PIMT in HUVEC at 48 h. D, F: Effects of GSPB2 on the expression of PIMT in HUVEC treated by gly-LDL at 48 h. GSPB2(L): 2.5umol/L; GSPB2(M): 5.0umol/L; GSPB2(H): 10.0umol/L. Data were expressed as the expression ratio of PIMT/β-actin and given as mean ± SD from three independent experiments. *P<0.05, **P<0.01 compared with CC group; ^#^ *P*<0.05, ^##^ *P*<0.01 compared with gly-LDL group.

We determined the effect of gly-LDL or high glucose on the expression of PIMT in HUVEC for 48 h by western blotting. Stimulation of HUVEC with different concentrations gly-LDL (0, 25.00, 50.00, 100.00 µg/mL) or D-glucose (0, 12.50, 25.00, 50.00 mmol/L) resulted in a significant decrease in the expression of PIMT (Figure 3A, 3B, 3C). Moreover, the expression of PIMT was significantly downregulated by stimulated of HUVEC with gly-LDL (50.00 µg/mL) or/and high glucose (25.00 mmol/L, HG). The pretreatment of HUVEC with different concentrations of GSPB2 (2.5, 5.0, 10.0 µmol/L, GSPB2(L), GSPB2(M), GSPB2(H)) significantly restored the gly-LDL (50.00 µg/mL)-induced decreaseof PIMT levels at 48 h (*P*<0.05) (Figure 3D, 3E, 3F).

### Role of PIMT on Viability in HUVEC Treated by gly-LDL

**Figure 4:**
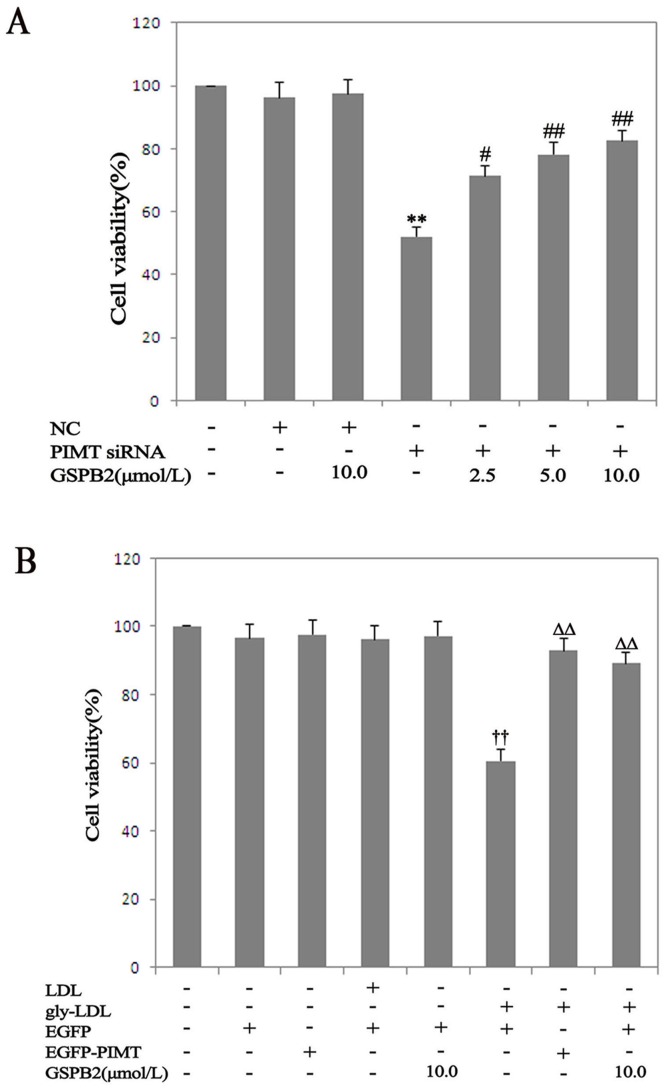
Effects of PIMT on cell viability treated by gly-LDL with MTT. A: Effects of PIMT siRNA, GSPB2 on viability in HUVEC treated by gly-LDL. B: Effects of PIMT overexpression, GSPB2 on viability in HUVEC treated by gly-LDL. Results are expressed as percent of untreated cells (100%) and are given as mean ± SD from five independent experiments. *P<0.05, **P<0.01 compared with NC group; ^#^ *P*<0.05, ^##^ *P*<0.01 compared with siPIMT group. ^†^ *P*<0.05, ^††^ *P*<0.01 compared with EGFP group; Δ*P*<0.05, ΔΔ*P*<0.01 compared with EGFP+gly-LDL group. GSPB2: grape seed procyanidin B2.

To investigate the role of GSPB2, PIMT in HUVEC viability, we transfected HUVEC with PIMT siRNA and plasmids, and estimated the cell viability using MTT assay. Moreover, unmodified LDL, EGFP, and negative control siRNA did not affect cell viability. Meanwhile, siRNA against PIMT significantly decreased the cell viability (*P*<0.05). The cell viability was increased when siPIMT group was exposed to GSPB2 (2.5, 5.0, 10.0 µmol/L) for 48 h (*P*<0.05) (Figure 4A). Moreover, the overexpression of PIMT restored the cell viability compared with EGFP+ gly-LDL group for 48 h (*P*<0.05) (Figure 4B).

### Role of PIMT in Apoptosis of HUVEC Treated by gly-LDL and the Anti-apoptotic Effect of GSPB2

**Figure 5:**
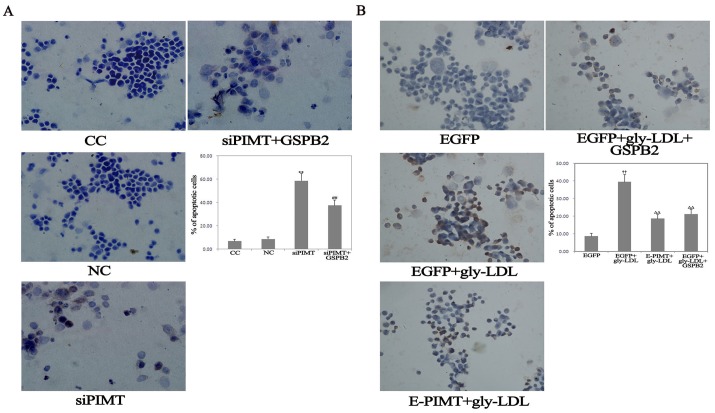
Effects of PIMT on cell apoptosis treated by gly-LDL with TUNEL assay (× 400). A: Effects of PIMT siRNA, GSPB2 on apoptosis in HUVEC. B: Effects of PIMT overexpression, GSPB2 on apoptosis in HUVEC treated by gly-LDL. The bar graph at the bottom shows the percentage of apoptotic cells. Results represent mean ± SD of five independent experiments. *P<0.05, **P<0.01 compared with NC group; ^#^ *P*<0.05, ^##^ *P*<0.01 compared with siPIMT group. ^†^ *P*<0.05, ^††^ *P*<0.01 compared with EGFP group; Δ*P*<0.05, ΔΔ*P*<0.01 compared with EGFP+gly-LDL group.

To examine whether PIMT plays a role in gly-LDL mediated apoptosis, we estimated the cell apoptosis using TUNEL. HUVEC transfected PIMT siRNA was susceptible to cell apoptosis, while GSPB2 (10.0 µmol/L) significantly attenuated the cell apoptosis for 48 h (Figure 5A). E-PIMT group and EGFP group were treated with or without gly-LDL (50.00 µg/mL) and treated with GSPB2 (10.0 µmol/L) for 48 h. Induction of EGFP group with gly-LDL (50.00 µg/mL) resulted in a significant increase in the cell apoptosis, whereas the overexpression of PIMT significantly attenuated gly-LDL treated the cell apoptosis compared with EGFP+ gly-LDL group (*P*<0.05). The treatment of EGFP+ gly-LDL group with GSPB2 (10.0 µmol/L) also significantly decreased the gly-LDL treated cell apoptosis for 48 h (*P*<0.05) (Figure 5B). These data clearly show that PIMT inhibited gly-LDL mediated cell apoptosis.

### Role of PIMT on Cytosol Cytochrome c Concentration, Caspase-9 and Caspase-3 Activity

**Figure 6:**
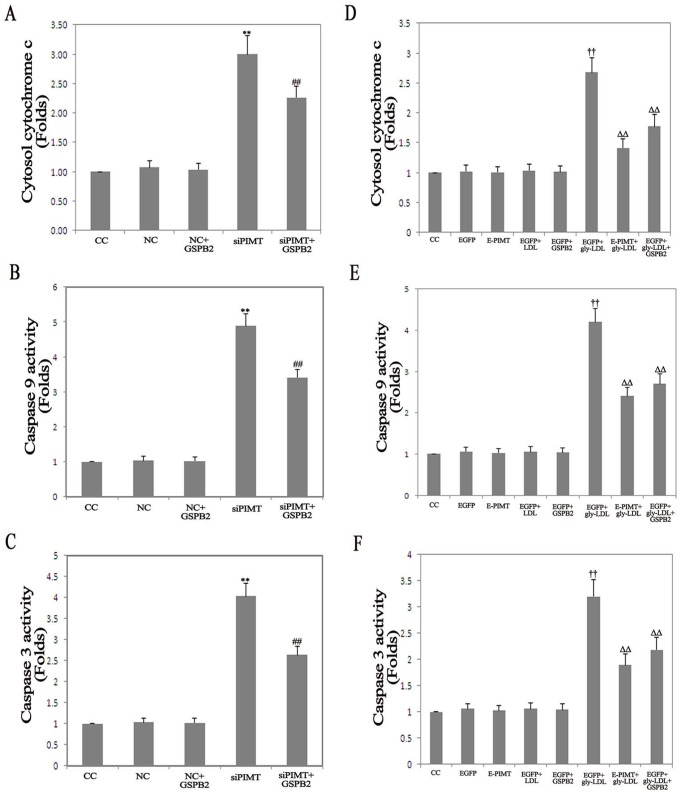
Effects of PIMT on cytosol cytochrome c concentration, caspase-9 and caspase-3 activity in HUVEC treated by gly-LDL. A, B, C: Effects of PIMT siRNA, GSPB2 on cytosol cytochrome c concentration, caspase-9 and caspase-3 activity in HUVEC. D, E, F: Effects of PIMT overexpression, GSPB2 on cytosol cytochrome c concentration, caspase-9 and caspase-3 activity in HUVEC treated by gly-LDL. *P<0.05, **P<0.01 compared with NC group; ^#^ *P*<0.05, ^##^ *P*<0.01 compared with siPIMT group. ^†^ *P*<0.05, ^††^ *P*<0.01 compared with EGFP group; Δ*P*<0.05, ΔΔ*P*<0.01 compared with EGFP+gly-LDL group.

To further examine the molecule mechanism of the apoptosis pathway, we examined the cytosol cytochrome c concentration, caspase-9 and caspase-3 activity induced by PIMT in HUVEC. The cytosol cytochrome c concentration, caspase-9 and caspase-3 activity were unchanged in NC group, NC+GSPB2 group, EGFP group, E-PIMT, EGFP+LDL and EGFP+GSPB2 group. The cytosol cytochrome c concentration, caspase-9 and caspase-3 activity significantly increased in siPIMT group compared with those in NC group, while GSPB2 (10.0 µmol/L) significantly inhibited the cytosol cytochrome c concentration, caspase-9 and caspase-3 activity in HUVEC of PIMT siRNA for 48 h (Figure 6A, 6B, 6C). Stimulation of EGFP group with gly-LDL (50.00 µg/mL) resulted in a significant increase in the cytosol cytochrome c concentration, caspase-9 and caspase-3 activity, whereas the overexpression of PIMT significantly attenuated gly-LDL treated the cytosol cytochrome c concentration, caspase-9 and caspase-3 activity compared with EGFP+gly-LDL group (*P*<0.05). The treatment of EGFP+gly-LDL group with GSPB2 (10.0 µmol/L) significantly improved the gly-LDL treated the cytosol cytochrome c concentration, EGFP+gly-LDL activity for 48 h (*P*<0.05) (Figure 6D, 6E, 6F). These results suggest that mitochondria pathway is involved in PIMT mediated endothelial cell apoptosis in response to gly-LDL treatment.

### Role of PIMT on the Levels of p53

**Figure 7:**
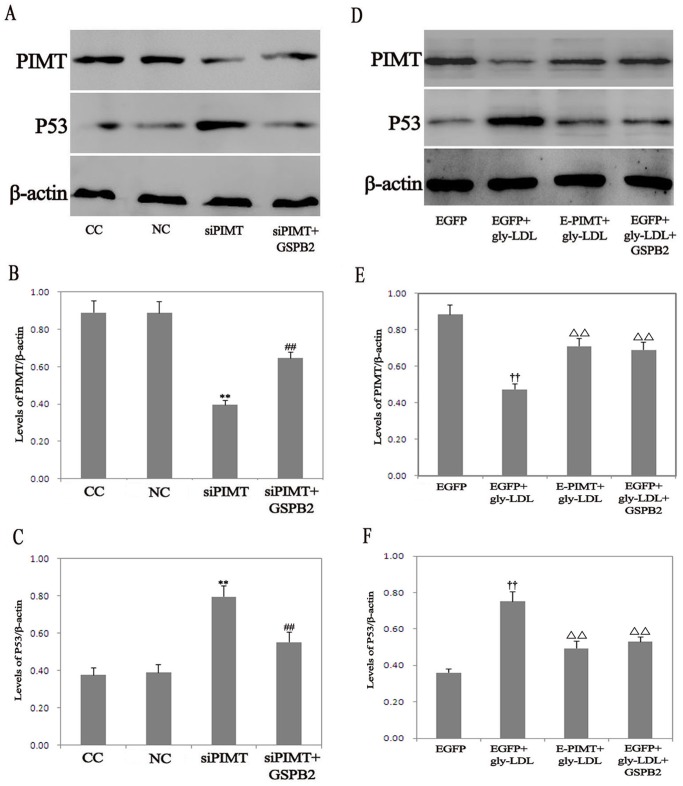
Effect of PIMT on the levels of p53 in HUVEC treated by gly-LDL. A, C: Effects of PIMT siRNA, GSPB2 on the expression of p53 in HUVEC. D, F: Effects of PIMT overexpression, GSPB2 on the expression of p53 in HUVEC treated by gly-LDL. B, E: Effects of GSPB2, gly-LDL on expression of PIMT in HUVEC. Data were expressed as the expression ratio of PIMT/β-actin, p53/β-actin and given as mean ± SD from five independent experiments. *P<0.05, **P<0.01 compared with NC group; ^#^ *P*<0.05, ^##^ *P*<0.01 compared with siPIMT group. ^†^ *P*<0.05, ^††^ *P*<0.01 compared with EGFP group; Δ*P*<0.05, ΔΔ*P*<0.01 compared with EGFP+gly-LDL group.

Given the well-established role of p53 in cell apoptosis, we determined the effects of PIMT on p53 in HUVEC. The level of p53 significantly increased in siPIMT group compared with NC group, while GSPB2 (10.0 µmol/L) significantly inhibited the expression p53 in HUVEC transfected PIMT siRNA (Figure 7A, 7C). The expression of PIMT in HUVEC exposed to gly-LDL was significantly downregulated for 48 h. Moreover, pretreatment of HUVEC with GSPB2 (10.0 µmol/L) significantly increased the expression of PIMT of HUVEC stimulated by gly-LDL (Figure 7B, 7E). Treatment of EGFP group with gly-LDL (50.00 µg/mL) resulted in a significant increase in the levels of p53, whereas the overexpression of PIMT significantly reversed the increased levels of P53 in response to gly-LDL. Treatment with GSPB2 (10.0 µmol/L) significantly attenuated the gly-LDL-induced increase of p53 level for 48 h (*P*<0.05) (Figure 7D, 7F).

### Role of PIMT on the Levels of phospho-ERK1/2 and phospho-GSK3β

**Figure 8:**
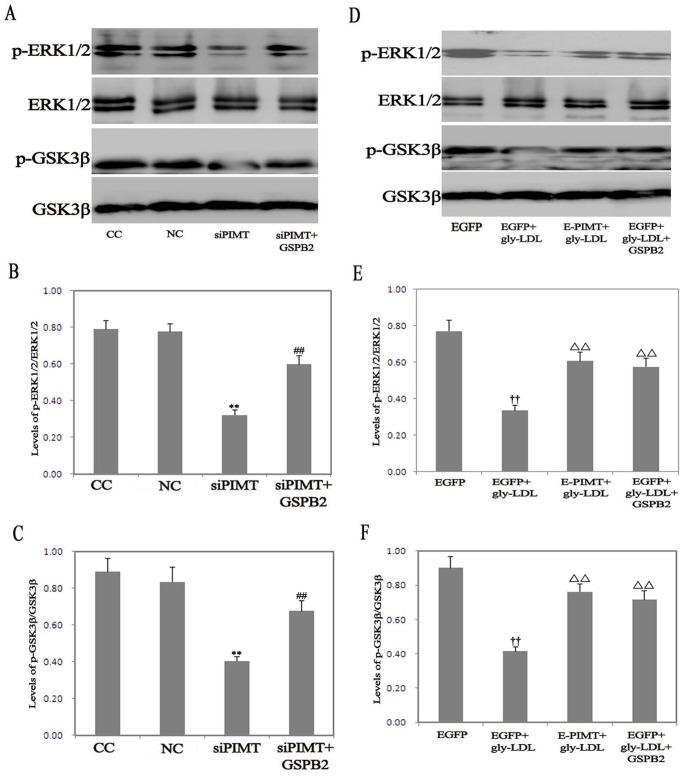
Effects of PIMT on phospho-ERK1/2 and phospho-GSK3β in HUVEC treated by gly-LDL. A, B, C: Effects of PIMT siRNA, GSPB2 on phospho-ERK1/2 and phospho-GSK3β in HUVEC. D, E, F: Effects of PIMT overexpression, GSPB2 on phospho-ERK1/2 and phospho-GSK3β in HUVEC treated by gly-LDL. Data were expressed as the expression ratio of phospho-ERK1/2/ERK1/2, phospho-GSK3β/GSK3β and given as mean ± SD from five independent experiments. *P<0.05, **P<0.01 compared with NC group; ^#^ *P*<0.05, ^##^ *P*<0.01 compared with siPIMT group. ^†^ *P*<0.05, ^††^ *P*<0.01 compared with EGFP group; Δ*P*<0.05, ΔΔ*P*<0.01 compared with EGFP+gly-LDL group.

We determined the effects of PIMT on phosphorylation of ERK1/2 (phospho-ERK1/2) and phosphorylation of GSK-3β (phospho-GSK3β) in HUVEC. The level of phospho-ERK1/2 and phospho-GSK3β significantly decreased in PIMT siRNA group compared with NC group, while GSPB2 (10.0 µmol/L) significantly reversed the decreased phospho-ERK1/2 and phospho- GSK3β in HUVEC transfected PIMT siRNA (Figure 8A, 8B, 8C). Treatment of EGFP group with gly-LDL (50.00 µg/mL) resulted in a significant decrease in the levels of phospho-ERK1/2 and phospho-GSK3β, whereas the overexpression of PIMT significantly reversed the decreased levels of phospho-ERK1/2 and phospho-GSK3βin response to gly-LDL. Treatment with GSPB2 (10.0 µmol/L) significantly attenuated the gly-LDL-induced decrease of phospho-ERK1/2 and phospho-GSK3β level for 48 h (*P*<0.05) (Figure 8D, 8E, 8F).

## Discussion

There is a lot of evidence that endothelial dysfunction is closely connected to the development of diabetic microangiopathy and macroangiopathy. More insights into the exact mechanisms underlying endothelial dysfunction may lead to important treatment strategies which can significantly reduce the morbidity and mortality rate caused by endothelial dysfunction especially in diabetes patients. Although apoptosis is a natural phenomenon in all multicellular organisms, an increased and accelerated rate of apoptosis of endothelial cells is probably a crucial factor in diabetic vascular complications [20], [21]. Due to hyperglycemia, LDL glycation is increased in diabetic patients. A recent study showed that gly-LDL played an important role in endothelial dysfunction of diabetes [22]. Artwohl havedemonstrated that gly-LDL induced endothelial cell death through the induction of apoptosis [5]. However, little is known about the molecular mechanisms on gly-LDL induced endothelial cell apoptosis.

GSPB2 has potent anti-apoptosis and anti-nonenzymatic glycation effects [23], [24]. Our previous studies showed that GSPB2 has protective effects against early stage endothelial dysfunction in DM [25]. Treatment of HUVEC with GSPB2 significantly inhibited the cell apoptosis induced by AGEs [18]. In this study, our results showed that GSPB2 significantly decreased the gly-LDL-induced cell apoptosis for 48 h. These data clearly demonstrated that GSPB2 participated in the prevention of gly-LDL-induced apoptosis. Moreover, the protective effect of GSPB2 on gly-LDL-induced apoptosis was dose-dependent.

It is known that PIMT is able to protect from high levels of pro-apoptotic proteins-induced apoptosis [12]. Moreover, the overexpression of PIMT in Escherichia coli or drosophila melanogaster increases the lifespan and survival under the heat stress [26], [27]. PIMT plays a role in the repair and/or degradation of damaged proteins and involves in the pathogenesis of diabetes mellitus and atherosclerosis [9], [28], [29]. We demonstrated here that gly-LDL down-regulated PIMT in HUVEC. These results suggest for the first time, to the best of our knowledge, that PIMT is involved in the process of gly-LDL-induced apoptosis. The overexpression of PIMT significantly increased the cell viability and attenuated gly-LDL-induced apoptosis, whereas PIMT siRNA showed the opposite effects.

Western blotting showed that treatment with GSPB2 increased PIMT levels in HUVEC. We hypothesized that GSPB2 through the activation of PIMT, at least in part, played protective effects. Studies have shown that GSPB2 have antioxidant effects [30], [31]. Oxidative conditions have been considered as a way through which proteins become more susceptible to deamidation. It has recently been reported that PIMT expression is likely modulated by oxidative damage in brain [32]. PIMT is able to repair abnormal proteins in vivo in which racemized or isomerized Asp residues accumulate during protein aging or under conditions of cell stress [33]. Cimmino reported that the overexpression of PIMT is able to prevent apoptosis induced by an oxidative treatment in endothelial cells [34]. These studies including ours suggested that GSPB2 might play antioxidative role in cell survival and apoptosis by regulating PIMT. However, whether and how the PIMT activated by GSPB2 and the inhibitory effect of PIMT on VEC apoptosis is related to its antioxidative effects will be the topic of our future studies. Several pathways have been demonstrated in mediating cell apoptosis, such as death receptors, mitochondria and endoplasmic reticulum pathway. It is well known that the mitochondria pathway plays a pivotal role in cell survival and apoptosis, and that mitochondrial dysfunction is a critical event in the apoptotic process [35]. We demonstrated here that the PIMT siRNA may be resulted in an inappropriate increase in the cytosol cytochrome c concentration. Cytochrome c release is a key step in activation of caspase cascade for initiation of apoptosis. Caspases are a highly conserved cysteine protease family and involved in the mitochondria-dependent apoptotic pathway, resulting in cleavage of specific proteins [36], [37]. Cytochrome c binds to pro-apoptotic factors and forms an apoptosome [38]. Apoptosome recruits and activates caspase-9, which in turn activates the downstream caspases, such as caspase-3 [39]. Caspase-3 is one of the key executioners of apoptosis, and its activation is a good marker for apoptosis. Our study showed that the apoptosis of HUVEC induced by gly-LDL was at least in part through the mitochondrial pathway. GSPB2 and overexpression of PIMT inhibited HUVEC apoptosis from gly-LDL exposure by inhibition of cytosol cytochrome c concentration, caspase-9 and caspase-3 activity.

p53 is a potent tumor suppressor and regulates several cellular processes, including metabolic homeostasis, DNA repair, growth arrest, senescence and apoptosis [40]. Our study revealed the role of p53 in gly-LDL induced HUVECs apoptosis. PIMT siRNA and gly-LDL resulted in a significant increase in the levels of p53, whereas GSPB2 and overexpression of PIMT significantly reversed the increased levels of p53 in response to gly-LDL. Recent study also found that PIMT negatively regulates the tumor suppressor protein p53 by reducing the transcriptional activity of p53 [41]. As a nuclear transcription factor, p53 can directly act in the cytosol and mitochondria to promote apoptosis through transcription independent mechanisms. Moreover, the p53-BclXL-Bcl2 complexes induced permeabilization of the outer mitochondrial membrane, resulting in cytochrome c release [42], [43]. Thus, we speculate that PIMT plays a critical role in inhibiting the activity of p53, which in turn influences the release of cytochrome c, and caspase-9 and caspase-3.

Glycogen Synthase Kinase 3 is a proline-directed serine/threonine kinase originally identified as a regulator of glycogen synthase. Of the two closely related isoforms, GSK3α and GSK3β, GSK3β is inactivated by phosphorylation of the amino-terminal serine 9 residue. It has been shown that the physiological effect of p53 is governed by inactivation of GSK3β(pSer9 GSK3β), suggesting that GSK3β may play crucial role in the regulation of cell death and survival by modulating mitochondrial apoptotic cell death pathway [44], [45]. Active GSK3β increases the cytochrome c release from mitochondria, which in turn activates successively caspase-9 and caspase-3. In the present study, we found that GSPB2 and overexpression of PIMT significantly attenuated the gly-LDL-induced decrease of phospho- GSK3β. These evidences demonstrate that GSK3β may serve as an upstream signaling molecule in mitochondrial apoptotic pathway.

ERK1/2 activation is a pivotal pathway for integration and transduction of antiapoptotic signals elicited by the paracrine component of endothelial apoptosis [46]. Several studies indicated that ERK1/2 can modulate mitochondrial functions, particularly those associated with cell death [47], [48]. It was demonstrated an antiapoptotic effect of ERK1/2 [49]. Our study is the first to elucidate that gly-LDL inhibited phosphorylation of ERK1/2 in HUVEC through a PIMT dependent manner. In addition, GSPB2 can upregulated the level of phospho-ERK1/2. Despite the involvement of ERK1/2 in the mitochondria, little is known about the molecular mechanisms of apoptosis that allow the process. Our study provide a novel link between PIMT and ERK1/2, it may be valuable to understand the role of ERK1/2 in gly-LDL-induced apoptosis.

### Conclusions

Taken together, our study shows that PIMT plays a key role in endothelial cells apoptosis induced by gly-LDL. The up-regulation of PIMT inhibits cell apoptosis from gly-LDL exposure through mitochondrial pathway. Moreover, GSPB2 might have benefits in cell apoptosis by inhibition of PIMT. Approaches targeting PIMT including use of GSPB2 could potentially lead to new avenue in the early stage of diabetic endothelial dysfunction.
